# Sentence Repetition as a Tool for Screening Morphosyntactic Abilities of Bilectal Children with SLI

**DOI:** 10.3389/fpsyg.2017.02104

**Published:** 2017-12-06

**Authors:** Elena Theodorou, Maria Kambanaros, Kleanthes K. Grohmann

**Affiliations:** ^1^Department of Rehabilitation Sciences, Cyprus University of Technology, Limassol, Cyprus; ^2^Department of English Studies, University of Cyprus, Nicosia, Cyprus; ^3^Cyprus Acquisition Team, Nicosia, Cyprus

**Keywords:** screening, clinical marker, referral criterion, bilectalism, Cypriot Greek

## Abstract

The clinical significance of sentence repetition tasks (SRTs) for assessing children's language ability is well-recognized. SRT has been identified as a good clinical marker for children with (specific) language impairment as it shows high diagnostic accuracy levels. Furthermore, qualitative analysis of repetition samples can provide information to be used for intervention protocols. Despite the fact that SRT is a familiar task in assessment batteries across several languages, it has not yet been measured and validated in bilectal settings, such as Cypriot Greek, where the need for an accurate screening tool is urgent. The aims of the current study are three-fold. First, the performance of a group of (Cypriot) Greek-speaking children identified with SLI is evaluated using a SRT that elicits complex morphosyntactic structures. Second, the accuracy level of the SRT for the identification of SLI is explored. Third, a broad error analysis is carried out to examine and compare the morphosyntactic abilities of the participating children. A total of 38 children aged 5–9 years participated in this study: a clinical group of children with SLI (*n* = 16) and a chronological age-matched control group (*n* = 22). The ability of the children to repeat complex morphosyntactic structures was assessed using a SRT consisting of 24 sentences. The results showed that the SRT yielded significant differences in terms of poorer performance of children with SLI compared to typically developing peers. The diagnostic accuracy of the task was validated, since regression analysis showed that the task is sensitive and specific enough to identify children with SLI. Finally, qualitative differences between children with SLI and those with TLD regarding morphosyntactic abilities were detected. This study showed that a SRT that elicits morphosyntactically complex structures could be a potential clinical indicator for SLI in Cypriot Greek. The task has the potential to be used as a referral criterion in order to identify children whose language needs to be evaluated further. Implications for speech–language therapists and policy-makers are discussed.

## Introduction

Identifying and diagnosing children with specific language impairment (SLI) is characterized internationally by both clinicians and researchers as an exceptional challenge. The principal goal of the present study is to determine whether a sentence repetition task (SRT), which includes different morphosyntactic structures, can serve as an accurate screening task, and as such as a referral criterion, for the early identification of SLI in Cypriot Greek-speaking children. In the long term, this will ensure access to early and comprehensive assessment for individuals with SLI and their families. The study also aims to examine whether sentence repetition can yield differences between groups of language-impaired vs. non-impaired participants in terms of morphosyntactic errors.

Whilst language acquisition is one of the most robust, yet largely intrinsically driven, processes of early childhood (e.g., Lenneberg, 1967; Chomsky, 1986), not all children acquire language fully or even effortlessly. The term SLI is applied to children that exhibit a significant deficit in language ability and yet, display normal hearing, have non-verbal intelligence in the broad range of normal with no obvious signs of neurological damage or social-emotional deprivation (Leonard, 1998; Bishop, 2014). We acknowledge that there is no consensus regarding the criteria for classification and the related terminology (Bishop et al., 2016), but an in-depth discussion on this matter is beyond the scope of this paper; we will subsequently employ the term SLI, noting that the “S” part may be debatable. The description of deviant or inferior language ability in SLI is usually based on (i) characteristics of children's spontaneous speech output and (ii) children's performance on linguistic tasks tapping into different language components (such as morphology, phonology, syntax, semantics, and pragmatics as well as the lexicon). There is now increasing evidence to suggest that children with SLI can present with different patterns of impairment based on which modules of the language system are impaired or spared, hence the absence of homogeneity in the disorder (e.g., Leonard, 1998; van der Lely, 2003; Friedmann and Novogrodsky, 2008).

Sentence repetition (also referred to as “sentence recall and sentence imitation”) taps into an individual's ability to repeat the exact wording of what was just heard. In the more recent past, research interest has turned to the diagnostic accuracy of the task. Studies have revealed that sentence repetition is a good psycholinguistic indicator of SLI in that consistently high diagnostic accuracy levels have been shown. For English, the observed positive correlation between sentence repetition with a number of language tests that are used widely, such as the Preschool Language Scale-3 (Boucher and Lewis, 1997), the Receptive and Expressive One Word Picture Vocabulary Test (Brownwell, 2000), and the Sentence Recall Subtest of the CELF (Wiig et al., 1992), has led to the assumption that the task can be a clinical marker for language impairment (Chiat and Roy, 2008). The term “clinical marker” refers to a particular structure that denotes SLI and for the purposes of this study it will be used for a task that includes different structures in accordance with similar research in the field (e.g., Conti-Ramsden et al., 2001; Stokes et al., 2006; Riches et al., 2010; Leclercq et al., 2014). Building on previous research, Riches et al. (2010) claimed that a SRT serves as an important tool in the diagnostic process of SLI. However, it is imperative to highlight that its validity as a potential clinical marker has not yet been evaluated systematically and fully.

While widely incorporated in language assessment tests (Dockrell and Marshall, 2015), the diagnostic accuracy of SRT s has not been investigated for many languages, such as Greek, including the Cypriot variety spoken in the Republic of Cyprus. Kamhi et al. (1984) already suggested that sentence repetition might produce more robust effects than spontaneous speech, and Everitt (2009) showed that it predicts later expressive abilities. This proposition followed the observation that children control their language productions by avoiding complex structures that are hard for them during spontaneous conversation. Consequently, in line with Seeff-Gabriel et al. (2010), we take it that a repetition task can be informative in terms of providing the full picture of children's linguistic strengths and weaknesses.

During the last two decades, researchers have turned their interest to the diagnostic utility of the SRT and found that it is a good indicator of SLI, showing high levels of sensitivity and specificity for children speaking English (Conti-Ramsden et al., 2001), Cantonese (Stokes et al., 2006), French (Thordardottir et al., 2011; Leclercq et al., 2014), and dialects of English (Oetting et al., 2016). For example, Conti-Ramsden et al. (2001) investigated whether sentence repetition—along with a third person singular task, tense marking, and non-word repetition—could be a clinical marker for the identification of SLI in English. They found that the strongest marker among those examined was sentence repetition, with sensitivity and specificity values for sentence repetition at 90 and 85%, respectively.

A similar result was revealed by Stokes et al. (2006), who examined Cantonese-speaking children. Specifically, they found that sentence repetition can accurately differentiate children with SLI from their typically developing peers. Moreover, significant differences between a group of 20 children identified with SLI (aged 7.2–13.0) and two groups of typically developing children (chronologically matched and language-matched) were found by Briscoe et al. (2001). Furthermore, Botting and Conti-Ramsden (2003) investigated four groups of language-impaired children, including children with SLI, and concluded that sentence repetition discriminates children with SLI from the other groups, including typically developing children, better than non-word repetition and past tense tasks do.

Thordardottir et al. (2011) examined the accuracy levels in SLI identification for 5-year-old French-speaking children and showed that the SRT used was sensitive (86%) and specific (92%). Similarly, the accuracy of a SRT used by speech–language therapists for SLI identification in French was examined (Leclercq et al., 2014) and yielded high accuracy levels were yielded. In particular, the study showed that 97.1% of children with SLI and 88.2% of typically developing children were classified correctly. Riches et al. (2010) extended the populations under investigation in their study and examined three groups: a group of 14 adolescents with SLI (mean age: 15.3), a group of 16 autistic children who exhibited language impairment (mean age: 14.8), and a group of 17 typically developing adolescents (mean age: 14.4). The research demonstrated that sentence repetition serves as a sensitive marker for language impairments in both clinical populations, adolescents with SLI and autism spectrum disorder.

The importance of meaningful diagnostic accuracy levels is discussed by Komeili and Marshall (2013) who support that tests with high specificity and sensitivity can minimize misdiagnosis, in terms of both under- and over-diagnosis. A further issue comes to light concerning the discrimination power of the task regarding age. Children between 3–6 and 6–11 years of age were tested on a repetition task and the results suggested that the younger children with SLI can be accurately identified in contrast to older children (Vender et al., 1981). Those findings were confirmed by research indicating that sentence repetition could be a sensitive clinical marker for younger children whose language abilities are incomplete, rather than for older children (Devescovi and Caselli, 2007). In contrast, the inclusion of complex sentences in a repetition task by Riches et al. (2010) showed that language-impaired individuals are identified even when they are adolescents. Other salient outcomes are those of Poll et al. (2010), who showed that sentence repetition is a good clinical marker of SLI in young adults.

Additionally the *type* of sentences included in a SRT has generated much discussion in the literature. Bernstein Ratner (2000) early on suggested that “[s]entences constructed at a level slightly above that observed in the child's spontaneous speech are regularized in ways that reflect both the child's extraction of form and meaning and the child's linguistic capacity” (p. 293). She presupposes that for the construction of a task, researchers need to take into account not only the age of the children under investigation *per se*, but their language development stage as well. However, this is not always possible because for a considerable number of languages, no clear developmental trajectories are available regarding how children acquire sentence structures—and this includes Greek generally, and in particular the variety of interest in the current study, Cypriot Greek.

For the purposes of this study, complex morphosyntactic structures were selected for investigation under the assumption that children have already acquired simple structures. When sentences are long enough, the participant cannot simply copy them. As a result, they resort to the grammatical system in order to be able to repeat the sentences by processing, analyzing, and reconstructing their meaning. This can only happen if the participant has already acquired the grammatical structures (Marinis and Armon-Lotem, 2015), hence relatively long and complex sentences are used in a SRT. In other words, in order to repeat a sentence, a child has to know its syntax. Polišenská et al. (2015) confirmed that performance on sentence repetition depends on language ability and in particular, in the areas of morphosyntax and lexical phonology. However, a child will not repeat a sentence if it is not fully understood either (Vinther, 2002). Therefore, the grammatical structure needs to be acquired first in order to be comprehended and expressed.

The findings regarding the use of complex syntactic structures in SRTs are not surprising given the well-documented difficulties in using those structures in SLI (e.g., Leonard, 1998; van der Lely and Battell, 2003; Novogrodsky and Friedmann, 2006). Indeed, there are syntactic structures that are not easy to elicit (Seeff-Gabriel et al., 2010), such as question structures and passives, and consequently they have not yet been evaluated. Despite the known utility of the tasks regarding the elicited data, SRTs that include these structures have been subject to scant investigation (Riches et al., 2010).

### Some background on Cypriot Greek

The Greek-speaking Republic of Cyprus, as it is summarized in Theodorou and Grohmann (2015), is generally described as “diglossia” (reviewed in Rowe and Grohmann, 2013), where the sociolinguistically “high” variety is typically accepted to be Standard Modern Greek (SMG), whereas the “low” variety is the vernacular Cypriot Greek (CG), of which Greek Cypriot is a native speaker. As can be accepted, the differences between the two varieties go far beyond the obvious aspects language such as vocabulary, pronunciation, and prosody. Distinct differences between CG and SMG are lexical, phonetic, and (morpho)phonological properties of the language (a host of research since the seminal study of Newton, 1972). With regard to the morphosyntactic level are among others personal pronominal clitics, which precede the finite verb in SMG while CG employs enclisis in indicative declarative clauses (much work since Agouraki, 1997). For recent research on the syntax of CG-speaking children's (a)typical language development, see among others Theodorou and Grohmann (2012) on relative clauses and Grohmann (2014a) for a review on clitics.

Because of the complex linguistic situation in Cyprus, the language status of Greek Cypriot children in this study is referrer-to as “bilectals,” as by adopted Rowe and Grohmann (2013), a term that has been used by various other researchers in recent research on language acquisition and subsequent development (e.g., Kambanaros et al., 2013; Grohmann, 2014b; Antoniou et al., 2016; Theodorou et al., 2016; Grohmann et al., 2017). In this context, bilectalism is used to characterize the linguistic situation in Greek-speaking Cyprus: Children of Greek Cypriot parents, with CG-speaking family and friends, grow up with CG from birth and yet, are exposed to SMG from an early age. This usually comes first through children's programme on TV, for example, and later through formal language instruction and interaction in public schools in all levels in SMG (though not necessarily in reality, as shown in Sophocleous, 2011; see also Leivada et al., 2017), thus enforcing exposure to SMG in a systematic way. Consequently, we further believe that language development in a bilectal context differs from very early on (Taxitari et al., 2015, 2017), both from monolinguals and bilinguals (Antoniou et al., 2016; Grohmann and Kambanaros, 2016).

The identification of language-impaired children in bilectal settings is not straightforward, since there are no screening or assessment tools specifically designed to diagnose impaired language in children who are CG-speakers (Kambanaros and Grohmann, 2013; Theodorou et al., 2016). Speech and language therapists (SLTs) as well as researchers usually rely on informal assessment measures, spontaneous language sampling, and clinical judgment to support the diagnostic process when formal diagnostic practices are not in place, a common phenomenon across a large number of EU countries (see Thordardottir, 2015). The diagnostic procedure becomes difficult not only because of the absence of appropriate screening and diagnostic tools for CG, it also creates confusion among policy-makers, teachers, and clinicians who may conceptualize both the language impairment itself and the need for speech and language services differently (Kambanaros and Grohmann, 2013).

In a more recent study (see also Theodorou, 2013; Theodorou et al., 2013), Theodorou et al. (2016) examined a number of norm-referenced tests published for SMG that assess the language abilities of monolingual children in Greece. These tests were modified into CG to address dialectal differences. The full assessment battery included measures of receptive vocabulary, comprehension and production of morphosyntax, metalinguistic concepts, sentence repetition, narrative retelling, articulation and phonological processing, word definitions, sound distinctions, and word finding. The study suggests that a combination of existing diagnostic tools support the diagnostic procedure when modified for CG on the basis of acceptable accuracy levels. This in turn allows the assumption that, if clinicians adopt the combinations suggested in that study, the likelihood for a correct diagnosis increases. The importance of accurate detection reflects on appropriate intervention, which has been acknowledged by several researchers (Fey and Cleave, 2008; Gallagher and Chiat, 2009).

This study addresses the question whether a SRT that elicits complex syntactic structures can serve as an accurate screening task for the identification of children who need further language assessment. Secondly, it will be evaluated whether there are qualitative differences in terms of morphosyntactic errors produced by children.

## Methods and materials

### Participants

Participants were 38 CG-speaking children aged 5–9 years who completed a SRT as part of a larger study about diagnosis of SLI in CG (e.g., Theodorou and Grohmann, 2015; Theodorou et al., 2016). The children were divided into four groups. Nine children were included in the younger group of children with SLI (SLI-Y: 7 boys and 2 girls, mean age 5.6, *SD* 0.3), and seven in the older group (SLI-O: 3 boys and 4 girls, mean age 7.8, *SD* 0.8). Ten participants were included in the younger group of TLD children (TLD-Y: 6 boys and 4 girls, mean age 5.8, *SD* 0.6) and twelve in the older group (TLD-O: 6 boys and 6 girls, mean age 7.10, *SD* 0.6). Building on our previous work (Theodorou et al., 2016), we compare the two groups of children with SLI to chronological age-matched groups following the proposed practice in assessing the accuracy of clinical markers (Plante and Vance, 1994; Bortolini et al., 2002, 2006). The background information on the 38 participating children is reported in Table 1.

**Table 1:** Participant details.

**Group**	**Age range**	**Number of participants**	**Mean**	**Stand. dev**.	**Gender**
TLD-Y	4.5–6.6	10	5.8	0.6	6M, 4F
TLD-O	6.7–8.7	12	7.10	0.6	6M, 6F
SLI-Y	4.11–5.11	9	5.6	0.3	7M, 2F
SLI-O	6.7–8.1	7	7.8	0.8	3M, 4F

*TLD, children with typical language development; SLI, children with specific language impairment; Y, younger; O, older*.

Subject selection criteria included: (i) CG-speaking background, (ii) no history of neurological, emotional, developmental, or behavioral problems, (iii) hearing and vision adequate for test purposes, (iv) performance within a broad range of normal on a measure of non-verbal intelligence (Raven's Coloured Progressive Matrices, Sideridis et al., 2015), and (v) no gross motor difficulties. All information was obtained either from speech therapists and teachers or from their parents. The children came from families with a medium to high socioeconomic status as measured by mother's education level using the European Social Survey (2010) database. Background information on the participating children is reported in Table 2.

**Table 2:** Participants' details.

**Group**	**Age range**	**No. of participants**	**Mean (*SD*)**	**Sig. (2-tailed)-Age**	**Gender**	**Mo's ed. (SD)**	**Sig. (2-tailed)-Mo's ed**.
TLD	4.5–8.7	22	6.10 (1.3)	0.29	12M, 10F	3.95 (1.1)	0.06
SLI	4.11–8.1	16	6.2 (1.3)		10M, 6F	3.37 (0.69)	

^*^ The mean difference is significant at the 0.05 level. SD, standard deviation; TLD, children with typical language development; SLI, children with specific language impairment; Y, younger; O, older; M, male; F, female; Mo's ed., mother's education (0 = did not complete primary education, 1 = completed primary education, 2 = competed high school, 3 = completed lyceum, 4 = diploma, 5 = university degree, 6 = master qualifications, 7 = PhD qualification).

Adopting the notion of “(discrete) bilectalism” from Rowe and Grohmann (2013), we consider “monolingual” children in diglossic speaker communities to be (at least) bilectal in the “high” and “low” varieties (see Kambanaros et al., 2013 for the first published study on child language implementing this term). With respect to the children participating in the present study, however, we can confidently state that they were all bilectal in CG (the native variety, spoken at home) and SMG (introduced formally in preschool; language of media and communication)—as understood through the works just cited. In particular, no children were simultaneous or sequential acquirers of an additional language and no child was a native speaker of SMG or received, to the best of our knowledge, any more input of strict SMG than any other.

Table 3 illustrates the performance of the children on the Raven's Progressive Matrices test (non-verbal IQ test) (Raven et al., 1998; Sideridis et al., 2015). Subject selection criteria included normal performance on the non-verbal IQ test. This requirement is satisfied for each child separately and there are no statistically significant differences in non-verbal IQ between the SLI groups and the controls.

**Table 3:** Means, standard deviations, and significant levels of all groups (Raven's).

	**Mean scores (standard deviation)**		**Sig. (2-tailed)**
**Groups**	**TLD (*****n*** = **10)**	**SLI (*****n*** = **9)**	
Younger	90 (12.47)	100.56 (12.86)	0.087
	**TLD (*****n*** = **12)**	**SLI (*****n*** = **7)**	
Older	94.58 (9.64)	95.71 (17.66)	0.880

*TLD, children with typical language development; SLI, children with specific language impairment*.

Children with SLI were recruited through private speech therapy clinics based on a protocol that included the previous identification of the participants by certified SLTs based on case history information, informal testing of comprehension and production, analysis of spontaneous language samples, and clinical observation. The diagnosis was later confirmed by a battery of tests developed for the assessment of SLI in Cyprus (Theodorou et al., 2016). The full assessment battery included measures of receptive vocabulary, comprehension and production of morphosyntax, metalinguistic concepts, sentence repetition, narrative retelling, articulation and phonological processing, word definitions, sound distinctions, and word finding. The groups' results on those tests are tabulated in Appendix A in Supplementary Material. The reader can find a detailed description of the recruitment procedure and complete descriptions of the tests in Theodorou et al. (2016).

### Sentence repetition task (SRT)

The ability of children to repeat syntactically complex sentences was assessed with an SRT, thus adopting the suggestion (Redmond, 2005; Stokes et al., 2006) that the stimuli of such a task should be complex in order to avoid ceiling performance. Accordingly, complex structures that are used frequently in CG, as in SMG were chose for inclusion. Indeed, it is important to note that for task construction and grading of structural difficulty, no model was adopted, because there is no relevant literature either for CG or for SMG. However, the items included represent structures that can be produced by typically developing children that are SMG speakers, as shown in corpora studies. Summing up, Mastropavlou and Tsimpli (2011) conclude that *na*-clauses can be produced even at the age of 2. Emergence of *pu*-relatives and *oti*-clauses follow later. Further, the structures included are those that have been found to be problematic for children with SLI either in Greek (including CG) (Stavrakaki, 2001; Theodorou and Grohmann, 2012) or in other languages, as the international literature (e.g., Leonard, 2001; Friedmann and Novogrodsky, 2004; Kunnari et al., 2014) suggests. The test consists of 24 items exploring the imitation of structures within six syntactic categories with four examples of each type: object relative clauses (1), subject relative clauses (2), embedded *oti* “that”-clauses (3), adjunct *giati* “because”-clauses (4), negative *den*-sentences (5), and subjunctive *na*-clauses (6).

1. Vlepo ti ^ŋ^gota pu a^ŋ^gaʎazi i ɣata.
  *I am watching the hen that the cat is hugging*.
2. Akouis to maθiti pu lali tin istoria.
  *You are hearing the pupil who is telling the story*.
3. Ipes oti i ʝaʝa emairepse su to fai.
  *You said that granny cooked your food*.
4. I daskala tu eçirokrotise ^n^don ʝati itan θcevazmenos.
  *His teacher applauded him because he was studious*.
5. O mixalis e ^n^do epline to proi.
  *Michalis didn't wash it in the morning*.
6. Prepi na mu to ðocis sto parko.
  *You must give it to me at the park*.

Specific language properties of CG were taken into consideration for the test design, including syntactic (e.g., clitics appear post-verbally: *eçirokrotise*^*n*^*don* in CG, *ton çirokrotise* in SMG), phonological (e.g., consonant deletion: *emairepse* in CG, *maʝirepse* in SMG), and morphological aspects (e.g., syllabic augment [e] in past tense: *eçirokrotise* in CG, ç*irokrotise* in SMG), among others (see Appendix B in Supplementary Material). The length of the sentences was between 9 and 13 syllables (mean: 15.54, *SD*: 4.34), which resembles sentences appearing in fairy-tales for pre-primary school level as well as the length of sentences appearing in text books grade 1. As for the vocabulary used, every day words and words that are frequently used in fairy tales and in the text books of grade 1 were selected, to avoid the vocabulary content having an undue influence on the sentence repetition ability (Polišenská et al., 2015). In particular, nouns and verbs were restricted to early-acquired words, such as “mum,” “granny,” “baby,” “food,” “want,” “say,” and “wash.”

### Procedure

The participants were asked to listen to 24 pre-recorded sentences. After each sentence, they were asked to repeat it as close to the original as possible. The stimuli were audio-recorded to ensure that all participants heard the sentences in the same way and presented via a PC in a fixed order using Power Point. The children were tested individually by trained research assistants. The examiner sat at a table either next to or opposite the children and said: “You are going to hear a sentence while you are watching the computer screen. You have to say exactly what you have heard.” On the computer screen a green circle would appear in order to keep the attention of the child away from other distractions in the room. No feedback was provided during the actual experiment, but encouragement was given when deemed necessary. Children's responses during the administration of the experimental task were audio-recorded using an Olympus WS-311M digital voice-recorder with a high-quality built-in microphone. These recordings were used to transcribe the children's responses for subsequent scoring.

### Scoring

Two different methods of scoring were examined. This decision was driven by Redmond's (2005) claim that in order for a task to be included in a battery aiming to detect SLI, a more refined scoring procedure is required. Consequently, the responses first were scored as correct (1 point) when a sentence was repeated exactly, with all the sentence elements included (hereafter Scoring Method 1). Scoring Method 1 mirrors that used for the TOLD-P3 Sentence Imitation subtest (Newcomer and Hammill, 1997) as well as the method adopted by Stokes et al. (2006) and Rispens (2004). Hence, the possible score range using this method was 0–24. For the second scoring method (hereafter Scoring Method 2), responses were scored according to the number of errors made in each sentence in agreement with the system developed for CELF-R (Semel et al., 1989), which was also used by Conti-Ramsden et al. (2001). That is, items were scored on a 0–3 scale, with 3 representing an exact repetition, 2 a sentence repetition with 1 error, 1 with 2 or 3 errors, and 0 with more than three errors. The maximum possible score using Scoring Method 2 was thus 72. For both scoring methods, phonological errors were not taken into consideration since the vast majority of the children with SLI exhibited some phonological difficulties as their performances for the phonological test indicate (see Appendix A in Supplementary Material). At this point, it is important to clarify that phonological processes used by our participants did not interact with calculated errors. For example, a common phonological process used was syllable deletion in multisyllabic words (e.g., [*epakolu*θ*usan*] instead of /*eparakolu*θ*usan*/ “they were watching”).

### Error analysis

In order to get some qualitative insights with regards to the morphosyntactic errors made by the participants a broad error analysis was followed. That is, each of the sentences produced was classified as syntactically correct either identical to the prompt or not. Then the errors or alternatives provided were classified as omission (7), substitution (8), addition (9), and change of word order (10) (Note that if the substitution resulted due to a phonological process only, it was not considered an error). A more detailed analysis followed to determine the affected linguistic element. Specifically, whether the error concerned a content word (7), free-standing morpheme (8), or an inflectional grammatical morpheme (11).

*Target sentence:* Vlepo tin ^ŋ^gota pu a^n^galiazi i γata.
  “I am watching the hen that the cat is hugging.”
Produced sentence:
  (7) Vlepo tin (omission/content word) pu a^n^galiazi i γata.
  (8) Vlepo tin ^ŋ^gota **na** (substitution/free-standing morpheme) a^n^galiazi i γata.
  (9) Vlepo tin ^ŋ^gota pu **tin** (addition/free-standing morpheme) a^n^galiazi i γata.
  (10) Tin ^ŋ^gota **vlepo** (change of the word order) pu a^n^galiazi i γata.
  (11) Vlepo tin ^ŋ^gota pu a^n^galiaz**e** (substitution/free-standing morpheme) i γata.

## Results

### Group differences

The performance of the four groups was compared according to the two scoring methods, provided in Table 4.

**Table 4:** Group performances on the SRT.

**Scoring method**	**Group**	**Mean**	***SD***
1 (out of 24)	TLD-Y	14.6	3.098
	TLD-O	18.2	4.366
	SLI-Y	7.9	3.790
	SLI-O	11.0	5.164
2 (out of 72)	TLD-Y	57.6	5.777
	TLD-O	63.5	7.379
	SLI-Y	40.2	13.890
	SLI-O	49.9	9.668

*TLD, children with typical language development; SLI, children with specific language impairment; Y, younger; O, older*.

**Figure 1:**
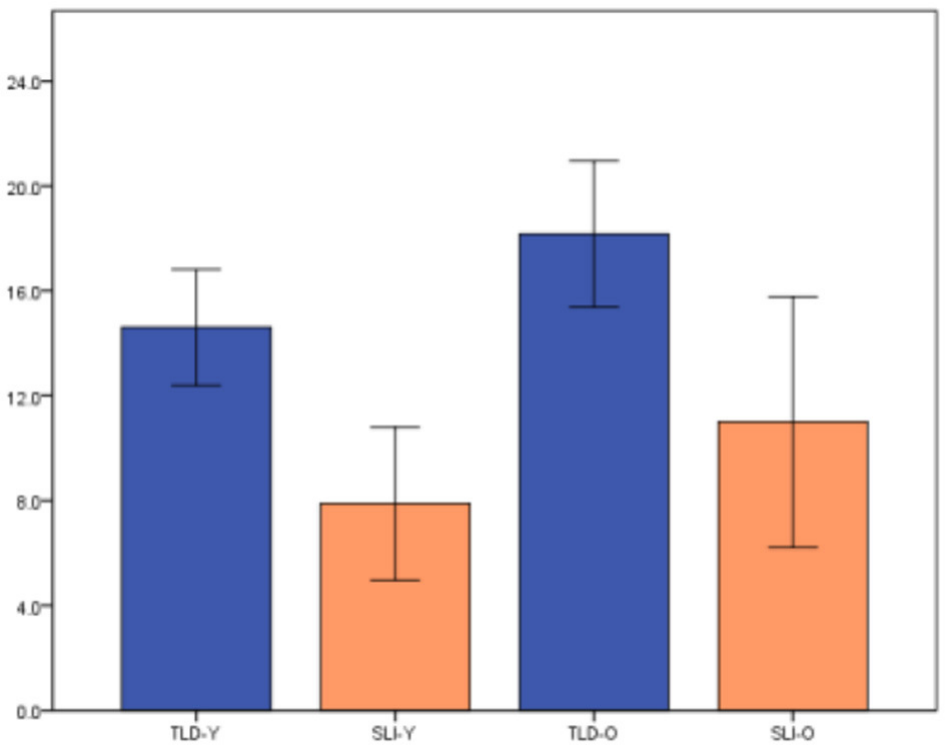
Significant differences for Scoring Method 1.

**Figure 2:**
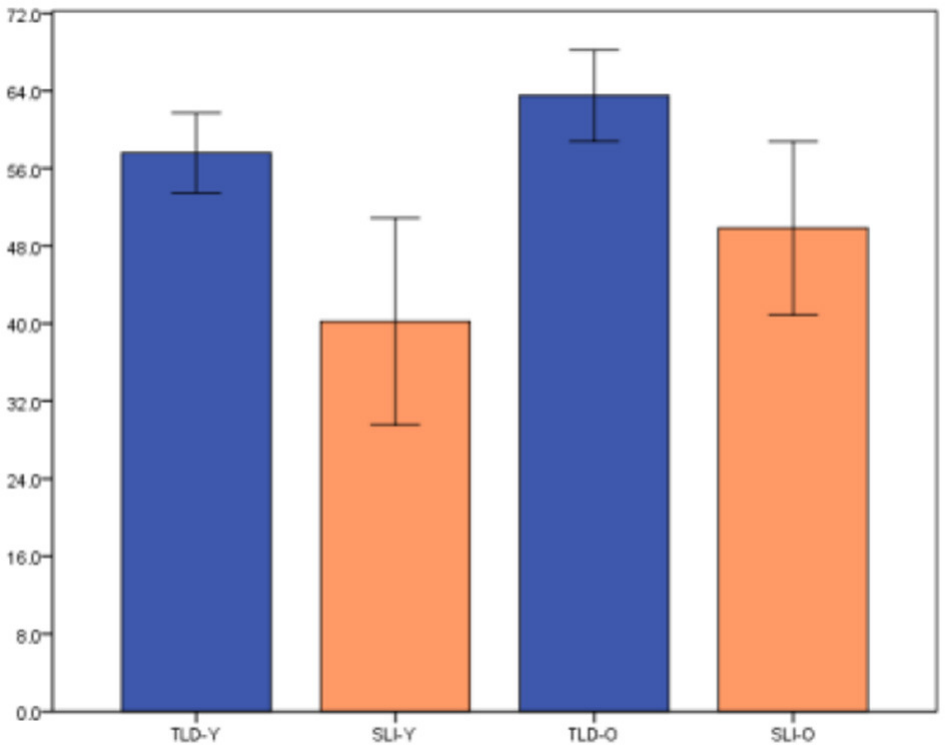
Significant differences for Scoring Method 2.

The differences on performance between children with SLI and TLD peers, with SLI scoring lower than TLD for both scoring methods, is graphically depicted in Figure 1 (Scoring Method 1) and Figure 2 (Scoring Method 2). To examine whether the task yielded significant differences between the groups, a one-way ANOVA was conducted. The test revealed significant differences between the groups for both methods, Scoring Method 1 [*F*_(3, 34)_ = 11.92, *p* = 0.00] and Scoring Method 2 [*F*_(3, 34)_ = 11.47, *p* = 0.00].

A two-way ANOVA was conducted to examine the effects of age (Old vs. Young) and language group (TLD vs. SLI) on the two scoring methods. For the first scoring method, both the main effect of age [*F*_(1, 34)_ = 6.072, *p* = 0.019] and the main effect of language group [*F*_(1, 34)_ = 26.226, *p* < 0.001] were significant. These results indicate that the TLD participants (*M* = 6.10, *SD* = 1.3) performed significantly higher than the SLI participants (*M* = 6.2, *SD* = 1.3). A non-significant interaction [*F*_(1, 34)_ = 0.028, *p* = 0.867] implies that the effect of language group was the same across the old and young participants.

**Figure 3:**
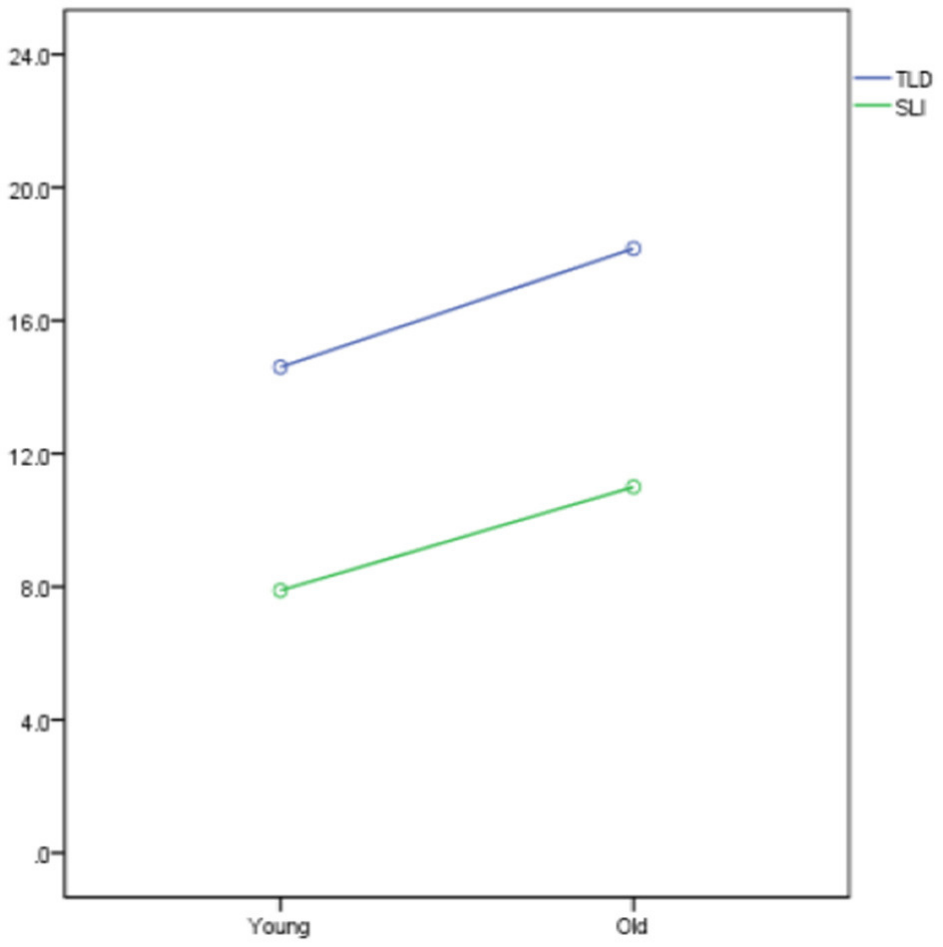
Interactions for Scoring Method 1.

**Figure 4:**
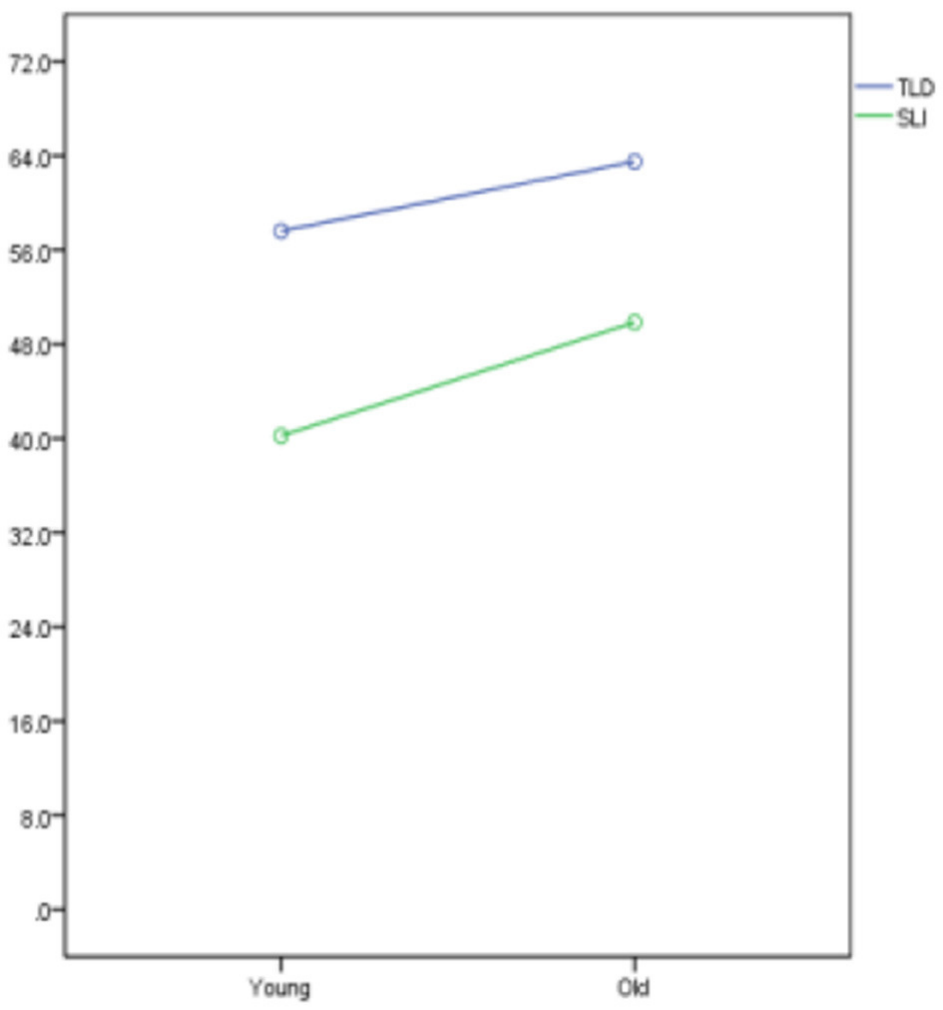
Interactions for Scoring Method 2.

Similar results apply for the second scoring method. Both the main effect of age [*F*_(1, 34)_ = 6.247, *p* = 0.017] and the main effect of language group [*F*_(1, 34)_ = 24.907, *p* < 0.001] were significant and their corresponding interaction was not significant [*F*_(1, 34)_ = 0.361, *p* = 0.552]. Again, the TLD participants (*M* = 6.2, *SD* = 1.3) performed significantly better than the SLI participants (*M* = 6.10, *SD* = 1.3) and the effect of language group was the same across the old and young participants. Interactions for scoring method 1 and scoring method 2 are illustrated in Figures 3, 4, respectively.

Summarizing so far, in line with other studies, CG-speaking children with SLI performed significantly below the TLD groups, rendering the SRT a potential clinical marker. Interestingly, the children's performance did not differ as a function of age, thus permitting the treatment of the participants as two groups, children with SLI and TLD children, for the remainder of the analysis.

### Specificity and sensitivity

It is already known that the significant differences between the groups are not reliable enough to characterize the SRT as an accurate tool for the detection of the impairment (Plante and Vance, 1994). Consequently, we proceeded to evaluate the sensitivity and specificity of the task used by conducting binary logistic regression analysis. More specifically, the analysis was carried out in order to show whether the children can be classified as children with SLI or TLD children, according to their performance in this task, for either of the two scoring methods or a combination of the two.

The results of the logistic regression analyses are tabulated in Table 5, where the percentages and the number of children that were correctly classified are shown for all three scoring arrangements.

**Table 5:** Percentages (and number of children) correctly classified by each scoring method.

**Scoring method**	**Children with SLI (sensitivity)**	**TLD children (specificity)**	**Overall accuracy**
1	12/16 (75%)	18/22 (81.8%)^*^	30/38 (78.9%)
2	12/16 (75%)	17/22 (77.3%)	29/38 (76.3%)
1 + 2	12/16 (75%)	18/22 (81.8%)^*^	30/38 (78.9%)

^**^ Good discriminant level,

* *Fair discriminant level*.

Scoring Method 1 seems to be more accurate than Scoring Method 2, whilst the combination of the two scoring methods reveals an identical accuracy level to Scoring Method 1. It appears that Scoring Method 1 can classify TLD children, as such, with 81.8% specificity, but it cannot classify SLI children equally well, as the reported sensitivity level is only 75%. Moreover, Scoring Method 1 can classify children with SLI at 78.9% accuracy. Summarizing so far, it is observed that Scoring Method 1 is an accurate discriminator for CG-speaking children with SLI, although the sensitivity level, in line with Plante and Vance (1994), cannot be characterized as adequate.

However, there is an issue that needs to be taken into consideration. One child belonging to the group of older children with SLI scored very high on this task, in contrast to his low performance in the other tasks, included in the diagnostic battery. This participant was a boy of 8.6 years who scored 22 out of 24 for Scoring Method 1 and 70 out of 72 for Scoring Method 2. His performance stands in stark contrast to the other children's performance included in the group, given the fact that the child whose performance followed his scored 12 and 53 on the two methods, respectively. Given this observation, we treated this particular child as an outlier and ran the regression analysis once more excluding him. Table 6 illustrates the percentages and the numbers of children that were correctly classified for each of the scoring methods as well for the combination of the methods as well, after the child was dropped from the analysis.

**Table 6:** Revised percentages (and number of children) classified by each scoring method.

**Scoring method**	**Children with SLI (Sensitivity)**	**TLD children (Specificity)**	**Overall accuracy**
1	12/15 (80%)^*^	18/22 (81.8%)^*^	30/37 (81.1%)
2	12/15 (80%)^*^	18/22 (81.8%)^*^	30/37 (81.1%)
1 + 2	11/15 (73.3%)	18/22 (81.8%)^*^	29/37 (78.4%)

^**^ Good discriminant level,

* *Fair discriminant level*.

It is interesting to note that the accuracy levels shifted slightly upwards. Table 6 shows that both scoring methods can classify accurately (81.1%) both groups, the children with SLI (sensitivity: 80%) and TLD children (specificity: 81.8%). However, with regards to the combination of the two methods, a slight reduction in the accuracy level is noted. A general outcome is that SRT can serve as a screening task for SLI identification. However, more research is needed, with more attention due to the design of the experiment.

### Morphosyntactic structures

**Figure 5:**
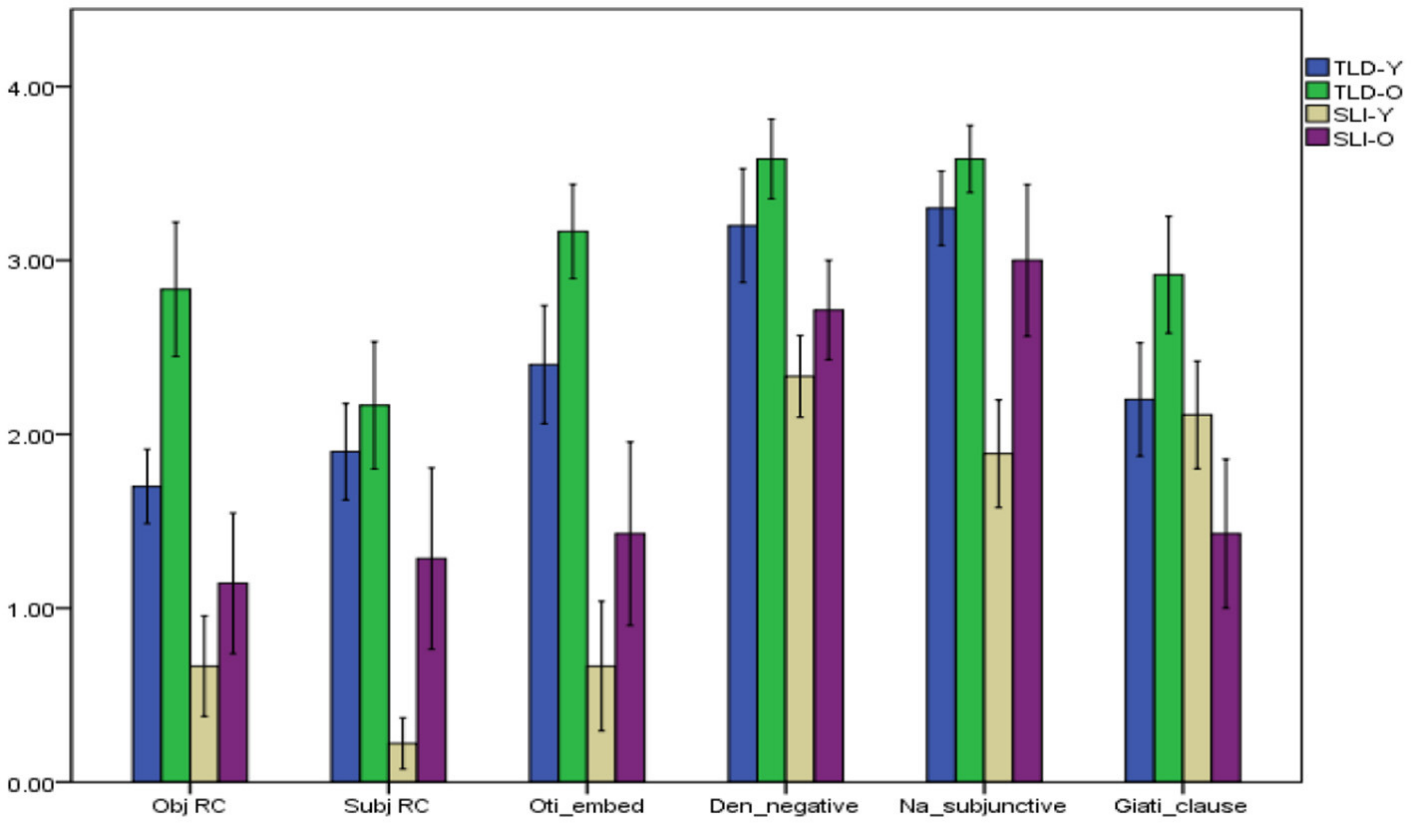
Sentence repetition in terms of grammatical structures.

The performance of children with SLI and their TLD peers in terms of correct raw scores on sentence repetition according to grammatical structure are graphically depicted in Figure 5 (individual results appear in Appendix C in Supplementary Material). It is observed that TLD children do not perform ceiling on the SR task. This is expected given that the stimulus included in the task are complex. Furthermore, and at least for research on relative clauses in CG (Theodorou and Grohmann, 2012), TLD children have not fully acquired them even at the age of 9 years old.

To examine whether significant differences yield between TLD children and children with SLI, *t*-tests were conducted. The analysis shows significant differences for the younger groups, between TLD-Y and SLI-Y, in object relative clauses [*T*_(17)_ = 2.918, *p* = 0.01], subject relative clauses [*T*_(17)_ = 5.178, *p* = 0.00], embedded *oti* “that”-clauses [*T*_(17)_ = 3.444, *p* = 0.003], negative *den*-sentences [*T*_(17)_ = 2.109, *p* = 0.05], and subjunctive *na*-clauses [*T*_(17)_ = 3.820, *p* = 0.001]. As for the older groups, significant differences were found between TLD-O and SLI-O in object relative clauses [*T*_(17)_ = 2.846, *p* = 0.011], embedded *oti* “that”-clauses [*T*_(17)_ = 3.259, *p* = 0.005], negative *den*-sentences [*T*_(17)_ = 2.342, *p* = 0.032], and adjunct *giati* “because”-clauses [*T*_(17)_ = 2.712, *p* = 0.015]. Analysis was carried out to examine whether significant differences were revealed between younger and older groups of children. A significant difference was detected between TLD-Y and TLD-O in terms of object relative clauses [*T*_(20)_ = −2.428, *p* = 0.025]. As for the comparisons between SLI-Y and SLI-O, analysis showed that there are significant differences in subject relative clauses [*T*_(14)_ = −2.191, *p* = 0.046] and subjunctive *na*-clauses [*T*_(14)_ = −2.138, *p* = 0.051].

### Error analysis

Acknowledging that sentence repetition allows for a collection of qualitative information about different language levels (Komeili and Marshall, 2013), for the purposes of the current study we investigate the errors made in terms of quantity. This is because of the main aim of the study, which is the evaluation of the SRT as a language-screening tool for CG-speaking children. Consequently, one of the scoring procedures followed by Stokes et al. (2006) was broadly applied, where the core elements of a sentence are isolated and then scored accordingly. First, the sentences produced were classified as syntactically correct or incorrect independently from the target sentences such as (12).

(12) Target sentence: Akuis to mathiti pu lali tin istoria.
  “You are listening to the pupil who is telling the story.”
Produced sentences: Akuis **ena** mathiti pu lali tin istoria.
  “You are listening to a pupil who is telling the story.”

A one-way ANOVA was conducted which shows significant differences between the groups [*F*_(3, 34)_ = 9.682, *p* = 0.00]. In order to find out whether there was a difference among the groups, a *post-hoc* Scheffé test was applied. The results show significant differences between younger children with SLI and younger TLD children (*p* = 0.004), whereas the difference between older children with SLI and older TLD children is not significant (*p* = 0.073).

**Figure 6:**
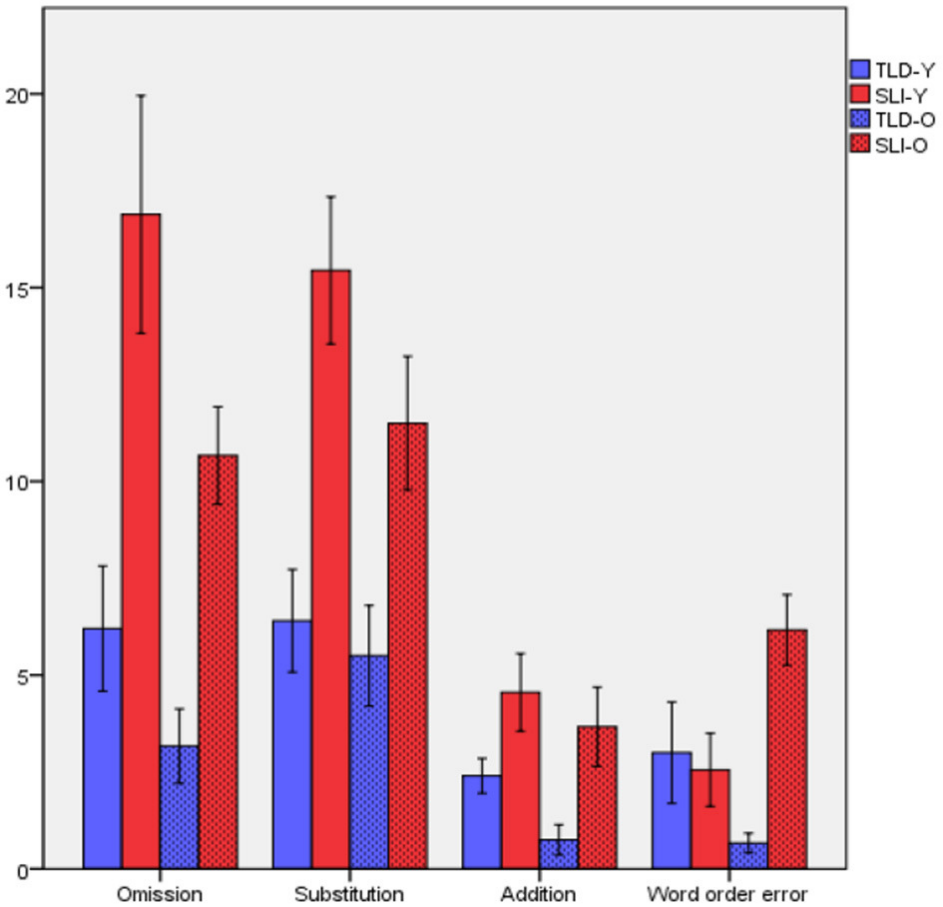
Distribution of errors made by the two groups of children.

Moving to a more detailed analysis, the errors made were classified as Omissions, Substitutions, Additions, and Word Order Error. As Figure 6 illustrates, differentiation between groups can be observed. To examine whether errors made yielded significant differences between the groups, a one-way ANOVA was conducted. The test reveals significant differences for all four types of errors [Omissions: *F*_(3, 34)_ = 10,059, *p* = 0.00; Substitutions: *F*_(3, 34)_ = 8,170, *p* = 0.00; Additions: *F*_(3, 34)_ = 5,732, *p* = 0.003; and Word Order Errors: *F*_(3, 34)_ = 3,864, *p* = 0.018].

In order to discover the groups that differ significantly, a *post-hoc* Scheffé test was conducted. Regarding Omissions, a significant difference was yielded between younger children with SLI and younger TLD children (*p* = 0.004) as well as between younger children with SLI and older TLD children (*p* = 0.000). Significant differences are also observed between younger TLD children and younger SLI (*p* = 0.004) and between younger SLI and older TLD (*p* = 0.001) in terms of Substitutions. In relation to Additions, the analysis shows significant difference only between younger children with SLI and older TLD children (*p* = 0.003). Moreover, older children with SLI differ significantly from older TLD children in terms of Word Order Errors (*p* = 0.02). It is highlighted here that no significant difference is detected between younger and older children in both cases, i.e., children with SLI and TLD children do not differ within the age groups for any of the error types.

Going a step further, we examined which morphological elements are affected in the produced sentences. To this end, the affected element—content word, free-standing morpheme, inflectional morpheme—was determined for each error. Table 7 presents the mean and standard deviation of the affected elements for each type of errors for all groups.

**Table 7:** Mean (standard deviation) of affected elements.

	**Omissions**			**Substitutions**			**Additions**			**Word order errors**		
	**Content word**	**Free-standing morpheme**	**Inflectional morpheme**	**Content word**	**Free-standing morpheme**	**Inflectional morpheme**	**Content word**	**Free-standing morpheme**	**Inflectional morpheme**	**Content word**	**Free-standing morpheme**	**Inflectional morpheme**
TLD-Y	2.1 (2.2)	4.2 (3.2)	0 (0)	2.4 (1.7)	3 (3.1)	1 (0.9)	0.2 (0.4)	2.2 (1.3)	0 (0)	1.2 (1.7)	1.8 (2.6)	0 (0)
SLI-Y	6.2 (4.0)	10.3 (5.1)	0.3 (1.0)	5.9 (3.1)	4.3 (1.9)	5.2 (3.8)	1.1 (1.2)	3.2 (2.3)	0.2 (0.4)	1.0 (1.5)	1.6 (1.5)	0.0 (0.0)
TLD-O	0.9 (1.2)	2.1 (1.9)	0.2 (0.6)	2.1 (1.8)	2.4 (2.1)	1.0 (1.3)	0.1 (0.3)	0.7 (1.4)	0.0 (0.0)	0.1 (0.3)	0.6 (0.8)	0.0 (0.0)
SLI-O	3.2 (2.8)	7.5 (2.1)	0.0 (0.0)	4.5 (1.2)	5.0 (2.4)	2.0 (2.0)	0.8 (1.2)	2.8 (1.5)	0.0 (0.0)	1.3 (1.0)	4.8 (1.6)	0.0 (0.0)

A one-way ANOVA was conducted to examine whether the affected elements are different for each group of participants. Significant differences were yielded between the groups for omission of content words [*F*_(3, 34)_ = 7.444, *p* = 0.001], omission of free-standing morphemes [*F*_(3, 34)_ = 10.515, *p* = 0.00], substitution of content words [*F*_(3, 34)_ = 6.117, *p* = 0.002], substitution of inflectional morphemes [*F*_(3, 34)_ = 7.902, *p* = 0.00], addition of content words [*F*_(3, 34)_ = 3.612, *p* = 0.023], addition of free-standing morphemes [*F*_(3, 34)_ = 4.326, *p* = 0.011], and change in the order of free-standing morphemes [*F*_(3, 34)_ = 5.375, *p* = 0.004]. The analysis continued with determining the pair of groups that differ significantly in terms of the affected morphological elements. They were found to differ significantly when a *post-hoc* Scheffé test was conducted. The results are provided in Table 8.

**Table 8:** Pairs of groups that differ significantly in terms of types of errors.

	**Pairs**	**Sign. level**
Omission of content word(s)	TLD-Y/SLI-Y	*p* = 0.017
	TLD-O/SLI-O	*p* = 0.001
Omission of free-standing morphemes	TLD-Y/SLI-Y	*p* = 0.006
	TLD-O/SLI-Y	*p* = 0.000
Substitution of content word(s)	TLD-Y/SLI-Y	*p* = 0.015
	TLD-O/SLI-Y	*p* = 0.005
Substitution of inflectional morphemes	TLD-Y/SLI-Y	*p* = 0.003
	TLD-O/SLI-Y	*p* = 0.002
	SLI-Y/SLI-O	*p* = 0.0041
Addition of content word(s)	SLI-Y/TLD-O	*p* = 0.046
Addition of free-standing morphemes	TLD-O/SLI-Y	*p* = 0.016
Word order error: Free-standing morphemes	TLD-O/SLI-O	*p* = 0.004

## Discussion

Research efforts on children with SLI have suggested sentence repetition capabilities can be a clinical marker. The primary interest regarding this study was to investigate whether SRT could serve as a screening task for bilectal CG-speaking children with SLI. The second aim was to identify the relation between SRT and a group of valid language tests included in a language assessment battery recently examined by the authors (Theodorou et al., 2016). Further analysis followed to examine the differences in terms of morphosyntactic errors produced by the participants.

Summing up, the SRT yielded significant differences in performance of CG-speaking children with SLI and those with TLD. The outcome confirms previous research findings for other languages, such as English (Conti-Ramsden et al., 2001; Seeff-Gabriel et al., 2010; Redmond et al., 2011), Cantonese (Stokes et al., 2006), Italian (Devescovi and Caselli, 2007), and French (Thordardottir et al., 2011; Leclercq et al., 2014), thus revealing that sentence repetition could be an effective clinical marker for bilectal CG-speaking children. We wish to highlight that the SRT used factored in dialectal (or variety) issues (Oetting et al., 2016) in the context of diglossia. Moreover, the majority of the grammatical structures used in the task was found to differentiate the performance of TLD children from their peers with SLI. This study is the first research to investigate sentence repetition in CG and therefore, further research is needed for a more complete picture.

The group differences found motivated the evaluation of the discrimination accuracy of the task. The high sensitivity and specificity levels which have been found for other languages, for example, English (Conti-Ramsden et al., 2001), are not replicated here, which may be due to the task design among other reasons that are discussed below. However, nearly accurate enough levels for Scoring Method 1 have been yielded (and slightly lower levels for Scoring Method 2).

Given the fact that sentence repetition has been found to be related to measures examining grammatical skills, namely, phonology, morphosyntax and semantics, an error analysis was conducted to compare the morphosytactic abilities of the participants. Our findings allow us to directly support the claim put forward in the relevant literature (Lust et al., 1996; Marinis and Armon-Lotem, 2015; Polišenská et al., 2015) that the performance on sentence repetition is an indicator of a child's grammatical ability.

Other noteable observations touch upon the errors made in terms of affected morphological errors-content words, free standing morphemes, inflectional morphemes. As for content words, though found to be affected, the differences between the groups are marginal, whereas more significant differences are observed for both free-standing and inflectional morphemes between the groupsInterestingly, no omission of inflectional morphemes was found which is arguably owed to the morphological richness of the Greek language where each lemma is usually highly inflected.

Another interesting revelation from the error analysis concerns the strategy of the older children with SLI (SLI-O) to produce alternative grammatically correct structures instead of the exact wording of what was heard. We can thus conclude that bilectal CG-speaking children with SLI do not produce ungrammatical sentences, but rather resort to structures that are accessible to them—even when considerably complex.

Summing up so far, the tool presented here could be adopted by SLTs as a screening task for identifying children who need further language assessment accurately. It is possible also for early education specialists (e.g., teachers) to be trained on the use and interpretation of the tool. This, in turn, would facilitate access to the appropriate services for language-impaired children. A short identification task would minimize the risk of non-identification and inaccessibility appropriate intervention, as has previously been recommended regarding evaluation protocols (Redmond et al., 2011).

The outcome of the task permits us to make a suggestion about the distinction of the discrimination power of the task in relation to the age of the children, in that younger children with SLI are differentiated more accurately than older ones (Vender et al., 1981; Devescovi and Caselli, 2007) has not been confirmed here. What is relevant is that older children with SLI produced syntactically correct sentences not identical to what they heard. The findings here tend to corroborate the suggestion by Riches et al. (2010) that SRT can identify older language-impaired children. It is assumed that the diagnostic accuracy has to do more with the type of the structures included in the task, rather than the task as such and is in agreement with Leclercq et al. (2014), who contend that SRT is very complex for children with SLI.

Apart from the matter of identification, some theoretical issues could also be addressed. Besides carrying out an analysis for both groups of TLD and language-impaired children, further analysis comparing younger and older groups did not reveal any significant difference. This outcome suggests that, at least for the set of structures included here, age does not play a role given that only minimal developmental progress is reported for children with SLI and for TLD children. Whilst the finding needs to be interpreted with caution, we contend that Greek Cypriot children, even at the age of 9, are still developing their language skills. As a consequence of this observation, we have insufficient evidence to make a definitive contribution to the ongoing debate pertaining to delay vs. deviance.

Additionally, researchers have highlighted several advantages of the task. First, it is claimed that SRT can be easily administered and analyzed (Lust et al., 1996), allowing for the evaluation of specific grammatical structures under controlled situations. That is, given the fact that it is implemented using a one-to-one format, this provides the opportunity for examiners to control the conditions in which children complete the task. In addition, a structured repetition task allows the investigator to select the target sentences carefully, according to the specific aims of the research, whereas this is not always possible if a spontaneous speech sample is evaluated. Thus, the researcher can examine morphosyntactic structures that are not easy to elicit either in spontaneous language or in other structured elicitation tasks. In addition, it is a natural skill that needs little effort and even young children recall sentences willingly. Moreover, it is postulated that the task does not seem to be influenced by factors, such as gender (Seeff-Gabriel et al., 2010). Concerning the relation between socioeconomic status and sentence repetition ability the existing evidence is contradictory, since there are studies that have contended there is a relation between high SES and better performance on SRT (Roy et al., 2014; Balladares et al., 2016), whilst others have reported no such influence (Gardner et al., 2006).

Some limitations of this investigation are reported as follows. First, the sample size is small and the age range quite large. However, sample size seems to be in line with the relevant published literature, such as Stokes et al.'s (2006) 16 and Seeff-Gabriel et al.'s (2010) 13 children with SLI investigated. Second, an issue that came to light concerns the construction of the task. We now believe that in the future, a replication of a tool to examine sentence repetition ability should take into consideration issues about language development and language impairment in CG (and SMG), such as structures that are expected to be developed by the ages under examination, rather than only the complexity parameter. By so doing, the task will become even more specific to structures that are documented as being problematic in the present study and previous research for CG (Theodorou, 2013; Theodorou et al., 2016). In addition, in order for the task to be administered for screening purposes, cut-off points should be established (Stokes et al., 2006), based on previous research Conti-Ramsden et al. (2001). Unfortunately, so far no standardized tests have been established for CG, although a battery of tests were found to be accurate in the diagnosis of SLI (Theodorou et al., 2016).

Another research direction could be the evaluation of SRT for measuring the progress of language intervention programs (Devescovi and Caselli, 2007). If there is evidence-based research that the SRT can really measure therapy progress, then the benefits will be two-fold. First, it could be a tool for SLTs to measure the effectiveness of the intervention. Second, policy-makers would then have tangible data to support the need for speech–language therapy services for those children with language difficulties. It is imperative to point out that the SRT presented here is not available to speech–language therapists yet, but a revised version could be in the future.

## Conclusion

It is crucial for clinicians and researchers alike to be sufficiently confident about the identification accuracy of a task used to identify children who experience SLI. However, no language test is able on its own to diagnose and describe the language abilities of a child in full and of course, none is sufficient to formulate recommendations for therapeutic intervention (Dockrell, 2001). Research has shown that sentence repetition is a useful tool for identifying children's language skills alongside other language tests. This study aimed to shed some light on the question whether children with SLI can be identified by using an SRT in the context of diglossia in Cyprus, where no diagnostic tests designed for the particular situation are available, and the results suggest such a task could be a potential clinical marker for SLI in CG. The outcome of this study is indicative and can be considered as a starting point for additional research.

## Ethics statement

This study was carried out in accordance with the recommendations of the Center of the Educational Research and Assessment of Pedagogical Institute of Cyprus with written informed consent from all subjects. All subjects gave written informed consent. The protocol was approved by the Center of the Educational Research and Assessment.

## Author contributions

All authors listed have made a substantial, direct and intellectual contribution to the work, and approved it for publication.

### Conflict of interest statement

The authors declare that the research was conducted in the absence of any commercial or financial relationships that could be construed as a potential conflict of interest.
