# Supplementary material for: Sentence Repetition as a Tool for Screening Morphosyntactic Abilities of Bilectal Children with SLI
Source: Front Psychol. 2017 Dec 6;8:2104. doi: 10.3389/fpsyg.2017.02104 (PMC5723908; doi:10.3389/fpsyg.2017.02104)
Supplement: Supplementary file 1 [file DataSheet1.docx]

**APPENDIX A**

Means, standard deviations, significance levels and effect sizes of the younger groups of bilectal participants (for all tests administered).

| TESTS | MEAN (SD) | | | Sig.  (2-tailed) | Effect size Cohen’s d |
| --- | --- | --- | --- | --- | --- |
|  | TLD–Y (n=10) | | SLI–Y (n=9) |  |  |
| ***Diagnostic Verbal IQ Test*** |  | | | | |
| Vocabulary | 22.9 (2.18) | 16.78 (2.82) | | 0.000* | 2.761 |
| Production: Morphosyntax | 19.8 (2.1) | 13.89 (2.71) | | 0.000* | 2.456 |
| Comprehension: Metaling. Knowl. | 19.9 (1.8) | 18 (3.87) | | 0.180 | 0.642 |
| Comprehension: Morphosyntax | 25.4 (2.6) | 24.56 (3.84) | | 0.578 | 0.259 |
| Sentence Repetitions | 45.50 (2.51) | 40.89 (2.47) | | 0.001* | 1.850 |
| TOTAL DVIQ | 133.50 (7.63) | 114.11 (10.45) | | 0.000* | 2.139 |
| ***Peabody Picture Vocabulary Test (Raw Scores)*** | 63.80 (11.73) | 54.78 (16.55) | | 0.185 | 0.635 |
| ***Expressive Vocabulary Test*** | 33.30 (5.14) | 21.67 (2.74) | | 0.000* | 2.779 |
| ***Bus Story Test*** |  | | | | |
| Information | 35.80 (11.54) | 21.78 (8.94) | | 0.009* | 1.348 |
| A5LS | 8.44 (2.12) | 5.40 (0.82) | | 0.001* | 1.852 |
| No. of Sub. Clauses | 7.80 (4.10) | 1.67 (1.5) | | 0.001* | 1.943 |
| No. of T-Units | 20.60 (3.89) | 15.56 (3.75) | | 0.011* | 1.318 |
| MLU | 4.70 (1.24) | 3.39 (0.70) | | 0.013* | 1.282 |
| ***ATHINA Test*** |  | | | | |
| Definitions | 15.44 (7.50) | 7.78 (2.39) | | 0.016* | 1.344 |
| Phonemic Discrimination | 18.40 (3.41) | 15.56 (6.29) | | 0.251 | 0.571 |
| ***Phonetic and Phonological Test*** | 66.9 (3.14) | 48.22 (9.76) | | 0.000* | 2.641 |

Key: TLD = Typically Developing children, SLI = children with Specific Language Impairment, SD = Standard Deviation, * = significant difference (p<0.05)

Note: Effect size indicates the difference in standardized values (>0.20 = small, >0.50 = medium, >0.80 = large, >1.30 = very large; Cohen, 1988))

Means, standard deviations, significance levels and effect sizes of the older groups of bilectal participants (for all tests administered).

| TESTS | MEAN (SD) | | Sig. (2-tailed) | Effect size Cohen’s d | |
| --- | --- | --- | --- | --- | --- |
|  | TLD-O (n=12) | SLI-O (n=7) |  |  | |
| ***Diagnostic Verbal IQ Test*** |  | | | | |
| Vocabulary | 24.67 (1.61) | 20.57 (1.81) | 0.000* | 2.436 | |
| Production: Morphosyntax | 21.33 (1.37) | 14.58 (1.9) | 0.000* | 4.279 | |
| Comprehension: Metaling. Knowl. | 22.58 (1.88) | 19 (1.73) | 0.001* | 1.958 | |
| Comprehension: Morphosyntax | 28.58 (1.38) | 26.43 (2.23) | 0.047* | 1.238 | |
| Sentence Repetitions | 47.33 (0.985) | 42.29 (2.36) | 0.000* | 3.130 | |
| TOTAL DVIQ | 144.50 (4.17) | 122.86 (6.31) | 0.000* | 4.302 | |
| ***Peabody Picture Vocabulary Test (Raw Scores)*** | 93.67 (25.87) | 72.86 (16.59) | 0.074 | 0.904 | |
| ***Expressive Vocabulary Test*** | 38.25 (3.70) | 27.71 (4.82) | 0.000* | 2.552 | |
| ***Bus Story Test*** |  | | | | |
| Information | 46.42 (8.87) | 29.00 (8.21) | 0.001* | | 2.016 |
| A5LS | 9.57 (2.36) | 7.86 (1.9) | 0.122 | | 0.774 |
| No. of Sub. Clauses | 9 (3.02) | 5.57 (1.9) | 0.015* | | 1.280 |
| No. of T-Units | 20.50 (3.32) | 20.14 (4.02) | 0.836 | | 0.100 |
| MLU | 5.24 (1.31) | 4.64 (1.14) | 0.326 | | 0.479 |
| ***ATHINA Test*** |  | | | | |
| Definitions | 21 (6.92) | 9.17 (0.98) | 0.000* | | 2.237 |
| Phonemic discrimination | 25.33 (3.5) | 16.71 (5.38) | 0.001* | | 2.024 |
| ***Phonetic and Phonological Test*** | 69.67 (0.49) | 66.14 (2.67) | 0.000* | | 2.160 |

Key: TLD = Typically Developing children, SLI = children with Specific Language Impairment, SD = Standard Deviation, * = significant difference (p<0.05)

Note: Effect size indicates the difference in standardized values (>0.20 = small, >0.50 = medium, >0.80 = large, >1.30 = very large

**APPENDIX B**

|  | **Structures** | **Dialectical characteristics** |
| --- | --- | --- |
| **object relative clauses** | | |
|  | Vlepo tin gota pu a^n^galiazi i γata.  ‘I am watching the hen that the cat is hugging.’ | Phonology |
|  | I Maria potizi ta louloudja pu efitepse i mama sto cipo.  ‘Maria is watering the flowers that her mum put in the garden.’ | Morphology |
|  | Citazo ti ^m^balarina pu ti zoγrafizi o zoγrafos.  ‘I am looking the ballerina that the painter is drawing” | Phonology |
|  | To peδaci troi ta γlika pu tu t aγοrasen o pap^h^us.  ‘The child is eating the sweets that the grandad bought him.’ | Syntax |
| **subject relative clauses** | | |
|  | Akuis to mathiti pu lali tin istoria.  ‘You are listening to the pupil that is telling the tale.’ | Morphology |
|  | Ta pedja vlepun ti jaja pu jirefci to pap^h^u.  ‘The children are watching the grand mum that is looking for the grandad.’ | Phonology |
|  | I aθlites angaljazoun tus γonis pu tus eparakoluθusan.  ‘The athletes are hugging their parents that were watching them.’ | Morphology |
|  | I kota tsi^m^ba to pulaci pu tis troi tin trofi.  ‘The hen is chicking the bird that is eating its food.’ | Phonology |
| **embedded *oti*-clauses** | | |
|  | Ipes oti i jaja emairepse su to fai.  ‘You said that the grand mum cooked the food for you’ | Phonology |
|  | O papas kseri oti etimasan da sto γrafio.  ‘The dad knows that they prepared them at the office.’ | Phonology, Syntax |
|  | O jatros ipen dus oti en:a jini gliora kala o arostos.  ‘The doctor said that the sick man will get well soon.’ | Phonology, Morphology, Syntax |
|  | Pcos anacinosen oti ta pedja en:a pan ekδromi.  ‘Who has announced that the children will go excursion?’ | Phonology, Morphology |
| ***dεn/mεn* negative clauses** | | |
|  | Men du tilefonisis.  ‘Don’t call him.’ | Phonology |
|  | O mixalis en to epline to proi.  ‘Michael didn’t wash it in the morning.’ | Phonology, Morphology |
|  | En tu lalo to mistiko tis.  ‘I don’t tell him my secret.’ | Phonology, Morphology, Syntax |
|  | I fili mu e mu to edoce piso otan tis to ezitisa.  ‘My friend didn’t give it back to me when I asked her.’ | Phonology, Morphology |
| ***na* + subjunctive clauses** | | |
|  | To moro fenete na aγapa pol:a ta pexniδca.  ‘The baby seems that it loves a lot the toys.’ | Phonology |
|  | I mama θeli na to fai.  ‘The mum wants to eat it.’ | Phonology |
|  | Prepi na mu to δocis sto parko.  ‘You have to give it to me at the park.’ | Phonology |
|  | I jaja θeli na tus to mairepsi avrio.  ‘The grandmum wants to cook it for them tomorrow.’ | Phonology |
| ***giati*-clauses** | | |
|  | To moro klei jati pina.  ‘The baby is crying because it is hungry.’ |  |
|  | To vazo espase jati erikse to kato i korua.  ‘The vase broke because the girl threw it.’ | Phonology |
|  | I δaskala tu exirokrotisen don jati itan θcevazmenos.  ‘The teacher applauded him because he was studied. ’ | Phonology, Morphology |
|  | To aγori efilaksen do jati θeli na tis to xarisi.  ‘The boy kept it because he want to offer it to her. ’ | Phonology, Morphology |

**APPENDIX C**

|  | ObjRC | SubjRC | *oti*-embedded clause | *dεn/mεn* negative clause | *na* + subjunctive | *giati*-clause |
| --- | --- | --- | --- | --- | --- | --- |
| TLD-Y1 | 2 | 3 | 2 | 4 | 4 | 3 |
| TLD-Y2 | 1 | 1 | 2 | 4 | 2 | 1 |
| TLD-Y3 | 1 | 1 | 4 | 4 | 3 | 2 |
| TLD-Y4 | 2 | 2 | 3 | 2 | 3 | 2 |
| TLD-Y5 | 1 | 1 | 1 | 4 | 4 | 1 |
| TLD-Y6 | 1 | 1 | 1 | 3 | 3 | 1 |
| TLD-Y7 | 2 | 2 | 3 | 4 | 3 | 3 |
| TLD-Y8 | 2 | 3 | 2 | 3 | 4 | 2 |
| TLD-Y9 | 2 | 3 | 4 | 3 | 3 | 3 |
| TLD-Y10 | 3 | 2 | 2 | 1 | 4 | 4 |
| TLD-O1 | 0 | 1 | 2 | 2 | 2 | 2 |
| TLD-O2 | 2 | 1 | 3 | 3 | 3 | 2 |
| TLD-O3 | 3 | 1 | 4 | 4 | 4 | 3 |
| TLD-O4 | 4 | 3 | 4 | 4 | 4 | 4 |
| TLD-O5 | 3 | 4 | 3 | 4 | 3 | 1 |
| TLD-O6 | 3 | 3 | 3 | 4 | 4 | 4 |
| TLD-O7 | 1 | 0 | 1 | 2 | 4 | 4 |
| TLD-O8 | 4 | 1 | 3 | 4 | 4 | 4 |
| TLD-O9 | 4 | 3 | 4 | 4 | 4 | 3 |
| TLD-O10 | 4 | 3 | 4 | 4 | 4 | 4 |
| TLD-O11 | 2 | 3 | 4 | 4 | 3 | 1 |
| TLD-O12 | 4 | 3 | 3 | 4 | 4 | 3 |
| SLI-Y1 | 0 | 0 | 0 | 1 | 0 | 1 |
| SLI-Y2 | 2 | 0 | 3 | 3 | 2 | 3 |
| SLI-Y3 | 0 | 0 | 0 | 2 | 3 | 1 |
| SLI-Y4 | 0 | 0 | 0 | 2 | 1 | 1 |
| SLI-Y5 | 1 | 1 | 0 | 2 | 2 | 3 |
| SLI-Y6 | 0 | 0 | 0 | 2 | 2 | 2 |
| SLI-Y7 | 1 | 1 | 2 | 3 | 2 | 3 |
| SLI-Y8 | 0 | 0 | 0 | 3 | 2 | 3 |
| SLI-Y9 | 2 | 0 | 1 | 3 | 3 | 2 |
| SLI-O1 | 1 | 2 | 1 | 4 | 4 | 1 |
| SLI-O2 | 1 | 1 | 0 | 2 | 3 | 1 |
| SLI-O3 | 1 | 1 | 2 | 2 | 1 | 1 |
| SLI-O4 | 2 | 0 | 1 | 2 | 2 | 1 |
| SLI-O5 | 0 | 0 | 0 | 3 | 3 | 1 |
| SLI-O6 | 3 | 4 | 4 | 3 | 4 | 4 |
| SLI-O7 | 0 | 1 | 2 | 3 | 4 | 1 |
